# A whole genome duplication drives the genome evolution of *Phytophthora betacei*, a closely related species to *Phytophthora infestans*

**DOI:** 10.1186/s12864-021-08079-y

**Published:** 2021-11-05

**Authors:** David A. Ayala-Usma, Martha Cárdenas, Romain Guyot, Maryam Chaib De Mares, Adriana Bernal, Alejandro Reyes Muñoz, Silvia Restrepo

**Affiliations:** 1 Research Group in Computational Biology and Microbial Ecology, Department of Biological Sciences, Universidad de los Andes, Bogotá, Colombia; 2 Max Planck Tandem Group in Computational Biology, Universidad de los Andes, Bogotá, Colombia; 3 Laboratory of Mycology and Plant Pathology (LAMFU), Department of Chemical and Food Engineering, Universidad de Los Andes, Bogotá, Colombia; 4 Institut de Recherche pour le Développement, CIRAD, Université de Montpellier, 34394 Montpellier, France; 5 Department of Electronics and Automation, Universidad Autónoma de Manizales, Manizales, Colombia; 6 Laboratory of Molecular Interactions of Agricultural Microbes (LIMMA), Department of Biological Sciences, Universidad de Los Andes, Bogotá, Colombia; 7 The Edison Family Center for Genome Sciences and Systems Biology, Washington University School of Medicine, MO 63108 St Louis, USA

## Abstract

**Background:**

Pathogens of the genus *Phytophthora* are the etiological agents of many devastating diseases in several high-value crops and forestry species such as potato, tomato, cocoa, and oak, among many others. *Phytophthora betacei* is a recently described species that causes late blight almost exclusively in tree tomatoes, and it is closely related to *Phytophthora infestans* that causes the disease in potato crops and other Solanaceae. This study reports the assembly and annotation of the genomes of *P. betacei* P8084, the first of its species, and *P. infestans* RC1-10, a Colombian strain from the EC-1 lineage, using long-read SMRT sequencing technology.

**Results:**

Our results show that *P. betacei* has the largest sequenced genome size of the *Phytophthora* genus so far with 270 Mb. A moderate transposable element invasion and a whole genome duplication likely explain its genome size expansion when compared to *P. infestans*, whereas *P. infestans* RC1-10 has expanded its genome under the activity of transposable elements. The high diversity and abundance (in terms of copy number) of classified and unclassified transposable elements in *P. infestans* RC1-10 relative to *P. betacei* bears testimony of the power of long-read technologies to discover novel repetitive elements in the genomes of organisms. Our data also provides support for the phylogenetic placement of *P. betacei* as a standalone species and as a sister group of *P. infestans*. Finally, we found no evidence to support the idea that the genome of *P. betacei* P8084 follows the same gene-dense/gense-sparse architecture proposed for *P. infestans* and other filamentous plant pathogens.

**Conclusions:**

This study provides the first genome-wide picture of *P. betacei* and expands the genomic resources available for *P. infestans*. This is a contribution towards the understanding of the genome biology and evolutionary history of *Phytophthora* species belonging to the subclade 1c.

**Supplementary Information:**

The online version contains supplementary material available at 10.1186/s12864-021-08079-y.

## Background

Pathogens of the genus *Phytophthora* are the etiological agents of many devastating diseases in a wide range of plant hosts, that include several high-value crops and forestry species such as potato, tomato, cocoa, and oak among many others [1, 2]. A notorious species of the genus is *P. infestans*, the causal agent of late blight of potato and tomato. This disease caused the Irish famine, a phenomenon that led to the exodus and death of millions of people in Ireland during the 19th Century [3, 4]. In the last decade, the southern region of Colombia has been affected by an increased incidence of late blight leading to devastating economic losses in tree tomato (*Solanum betaceum*) crops. Mideros et al. [5] described the novel species *P. betacei* as the causal agent of the mentioned late blight of the tree tomato, instead of the sympatrically occurring *P. infestans* that causes the disease in potato crops.

*Phytophthora betacei* species was erected and distinguished from *P. infestans* and its closely related species *P. andina* using morphological, physiological, molecular, and host-specificity evidence in isolates obtained from crops with late blight [5]. The molecular evidence, based on simple sequence repeat (SSR) and genotyping-by-sequencing (GBS) markers, allowed the classification of isolates previously classified as the EC-3 clonal lineage of *Phytophthora infestans sensu lato* (using RFLP data [6]) into the putative *P. betacei* species. Furthermore, the data from 22 788 SNPs revealed a clear genetic structure among the three evaluated species, except for two individuals (likely misclassified) of *P. andina* clustering within the EC-3 lineage along with *P. betacei* individuals. This suggested that *P. infestans, P. andina*, and *P. betacei* are genetic differentiated groups with negligible gene flow between them [5].

Although GBS data provides valuable genetic evidence to support speciation, there is no public genome assembly or sequencing data for *P. betacei* that allows for further inquiry. Also, the lack of public genome assemblies of *P. infestans* from Colombia and the Americas limits the research on genomic-scale evolution and regional genetic diversity of the late blight pathogens. Whole genome sequences are needed to perform structural and comparative genomics analyses aiming to explore the species diversification dynamics in these *Phytophthora* species.

The nuclear genome of *Phytophthora* species is highly plastic; the term “two-speed genomes” has been coined [7] for this genus, due to their evolutionary dynamics, in a manner similar to other filamentous plant pathogens. The term means that the core orthologous housekeeping genes of the individual are distributed in gene-dense regions under purifying selection, whereas its pathogenicity and virulence-related genes are distributed along gene-sparse regions with high content of transposable elements and repeats under positive selection [7, 8]. Some of these virulence-related genes, named effectors, are used by the pathogen to promote infection by modulating the immune response of the host. This is carried out by their interaction with apoplastic or cytoplasmic molecular targets and they may determine the pathogenicity in a susceptible host [9]. Effectors such as those from the RxLR and Crinkler (CRN) families translocate inside host cells [10, 11] and constitute two of the most studied virulence-related genes in the genus *Phytophthora* and tend to be located in gene-sparse regions [8, 12]. It has been observed that extensive gene duplication, gene loss, and genomic rearrangements have occurred in the genomes of *P. infestans*, *P. sojae*, and *P. ramorum*, particularly in gene-sparse regions where effector genes are located, and evidence points to bursts of lineage-specific transposon activation as the cause of such phenomena [8, 13].

Repetitive content in *Phytophthora* species, particularly in the gene-sparse regions described above, represents an important fraction of the total genomic content, accounting for up to 74 % of the sequenced bases in *P. infestans* T30-4 [12]. This repetitive content also represents a big challenge for accurate assembly with short-read sequencing technologies [14], as those used up to date, to reconstruct the representative assemblies of all members of the genus [2, 12, 15]. The emergence in recent years of long-read sequencing technologies such as PacBio SMRT or Oxford Nanopore sequencing has unveiled a whole new panorama for *Phytophthora* genomics. Indeed, by yielding single-molecule reads of thousands of base pairs in length per run in any of these platforms, there is a reduced chance of mis-assembling highly repetitive regions, as long as the repeat is shorter than the read length. An additional advantage of these platforms is preventing sub-representation of GC-rich and low-complexity genomic regions since they do not rely on PCR amplification of the source genomic material [16]. In summary, long read technologies pose the potential of yielding more accurate assemblies for the complex genomic architectures of the *Phytophthora* genus compared with previous short-read technologies.

Beside transposable elements, it has been reported that independent whole genome duplications (WGD) have shaped two closely related *Phytophthora* pathogens affecting Cacao: *P. palmivora* and *P. megakarya* [17, 18]. As a consequence of WGD and invasion of transposable elements, the genomes of *P. palmivora* and *P. megakarya* are particularly large (135 and 222 Mb). Mideros et al. [5] estimated the nuclear DNA content of *P. betacei* isolate P8084 to be almost twice as large (1.13 pg) as that of the reference strain *P. infestans* T30-4 (0.67 pg), thus raising the question of whether a transposon-driven genomic expansion or a WGD occurred independently in this lineage.

In this study, we perform genome assembly and annotation of *P. betacei* P8084 and a Colombian strain of *P. infestans* (RC1-10) using long-read SMRT sequencing technologies. The genome assembly size of *P. betacei* is about 270 Mb, the largest assembled genome in the *Phytophthora* genus. A moderate transposable element invasion and a WGD likely explain its genome size expansion when compared to *P. infestans*. This is the first approach towards the understanding of the genomic expansion and evolutionary history of this novel *Phytophthora* species.

## Results

### Genome sequencing, polishing, and haploid representation

The complete sequencing dataset for *P. betacei* P8084 was composed by ~ 17.5 Gb of uncorrected PacBio Sequel subreads combined with ~ 22.7 Gb of Illumina paired-end (PE) reads generated in a previous study [5]. For *P. infestans* RC1-10 only PacBio Sequel sequencing was carried out and the yield of the uncorrected subreads was ~ 9.2 Gb.

A k-mer coverage frequency analysis of the Illumina data obtained for *P. betacei* P8084 showed a very likely scenario for a highly-heterozygous diploid organism (heterozygosity rate = 3.51 %) (Supplementary Fig. 1), after which we performed the assembly using only long-reads and assuming both organisms to be diploid. To account for the high heterozygosity and the fact that this assembly is unphased, we performed haplotig curation. Afterwards, we used a two-step polishing approach to correct for indels and misassigned bases in the assemblies.

The final assembly statistics are shown in Table 1. We also included the publicly available reference genomes of the genus, namely *P. nicotianae, P. sojae*, and *P. ramorum* RefSeq assemblies, as comparison. The RefSeq genome of *P. infestans* T30-4 served as the gold standard. *Phytophthora betacei* P8084 has a genome size of 270 Mb, the largest of all the evaluated assemblies, distributed among 802 contigs. It was 42 Mb and 70 Mb larger than *P. infestans* T30-4 and *P. infestans* RC1-10 genomes, respectively, and almost 200 Mb (3.4X) larger than other *Phytophthora* genomes, constituting a more significant assembly challenge. The majority of the sequenced bases (98.3 %) were found in contigs larger than 50 kb. The contig number of *P. betacei* P8084 is one order of magnitude greater than *P. sojae*, within the same order of magnitude as *P. nicotianae*, and one order of magnitude lower than the other assemblies. The contiguity of *P. betacei* assembly, as measured by the N50, is ~100 kb, making it one order of magnitude less contiguous than *P. infestans* T30-4 and *P. sojae*, but equivalent to the rest of the genomes. *Phytophthora sojae* assembly metrics show the largest N50 value, the smallest contig number, and largest percentage of bases sequenced in contigs>=50 kb, however it should be considered that is approximately 1/3 of the genome size. *P. betacei* was placed second for two of such statistics with a significantly larger genome.

**Table 1:** Assembly statistics of *Phytophthora betacei* P8084 and *Phytophthora infestans* RC1-10 compared to *Phytophthora* RefSeq genomes

Assembly	*P. betacei* P8084	*P. infestans* (RC1-10)	*P. infestans* RefSeq (T30-4)	*P. sojae* RefSeq (V3 P6497)	*P. nicotianae* (P1569)	*P. ramorum* RefSeq (Pr102)
Contigs/Scaffolds	802	2902	4921	82	5010	2576
Total length (Mb)	270.89	203.29	228.54	82.60	55.10	66.65
Genome in contigs > = 50 Kb (%)	98.29	76.90	89.88	99.12	27.90	72.63
Largest contig (Mb)	4.15	0.88	6.93	13.39	0.30	1.24
GC (%)	51.00	51.00	51.00	55.00	49.60	54.00
N50 (Kb)	737.97	120.01	1588.62	7609.24	27.20	308.04
L50	103.00	413.00	38.00	4.00	549.00	63.00
N’s in the assembly (%)	0.00	0.00	16.81	3.96	0.00	18.35

See Methods section for the accession numbers of the RefSeq genomes in Genbank database

[This table should appear in the same page than the Results section named *Genome sequencing, polishing, and haploid representation*]

The *P. infestans* RC1-10 assembly has a total size of 203 Mb contained in 2902 contigs, being smaller than the gold standard T30-4 genome which contains 228.5 Mb. It was also more fragmented with only 76.9 % of the sequenced bases present in contigs larger than 50 kb compared to 89.9 % in T30-4. Both *P. infestans* assemblies have a higher contig count and lower values for bases present in large contigs, but the RefSeq assembly showed a much higher N50 than RC1-10. An important advantage of using long-read assembly is that despite the quality differences among the assemblies generated in this study, *P. betacei* P8084 and *P. infestans* RC1-10 genomes have 0 % N’s in the assembly, compared to 16.81 % in the *P. infestans* T30-4 RefSeq genome.

**Fig. 1:**
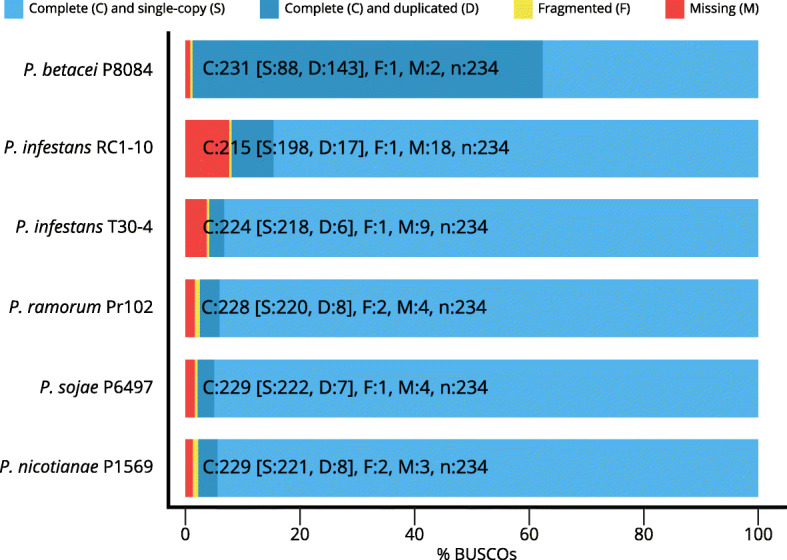
BUSCO assessment of *Phytophthora betacei* P8084, *Phytophthora infestans* RC1-10, and reference assemblies of the genus. See Methods for GenBank accession numbers

We also evaluated the semantic content of the genomes with BUSCO to have an estimate of gene recovery for each assembly. Of all the *Phytophthora* genomes evaluated for presence of single-copy orthologs in a Stramenopila-Alveolata dataset, *P. betacei* P8084 showed the fewest number of missing genes, followed by *P. nicotianae*. *Phytophthora betacei* P8084 also showed the highest number of duplicated genes of the evaluated panel. *Phytophthora infestans* RC1-10 showed a missing gene content greater than that of the other genomes but has comparable metrics of fragmented and duplicated genes relative to all the other species (Fig. 1). Together, these data suggest that *P. betacei* P8084 is one of the most semantically complete genomes of the genus, and it might have extensive duplication of core orthologs compared to all other compared assemblies. Also, it is a hint that *P. infestans* RC1-10 has an acceptable assembly quality comparable to public RefSeq genomes.

### Gene prediction and functional annotation

MAKER 2 gene prediction and annotation pipeline was executed for both *P. betacei* P8084 and *P. infestans* RC1-10 with the transcriptome and proteome of *P. infestans* T30-4 as evidence to support gene prediction. This pipeline was also run for *P. infestans* T30-4 in a similar way for comparison purposes. The prediction yielded a total of 23 457 genes for *P. betacei* P8084, 15 893 genes for *P. infestans* RC1-10 and 17 475 genes for *P. infestans* T30-4 with 14 929 (63.64 %), 9680 (60.91 %) and 10 506 (60.12 %) of the transcripts, respectively, having an assigned function as evidenced by domain identification via InterProScan or BLAST searches against UniRef90 database (Table 2). Uncharacterized proteins without assigned function represented between 36 and 39 % of the total predicted genes for all three genomes, although novel proteins that did not match any previously reported uncharacterized proteins comprised only ~ 2 % of the total gene content of each genome. In total, *P. betacei* P8084 showed a slightly larger proportion of genes relative to the sequenced genome size compared to both *P. infestans* assemblies (Table 2).

**Table 2:** Gene annotation statistics from MAKER2 and CAZymes for *Phytophthora betacei* P8084 and *P. infestans* assemblies

Assembly	*P. betacei* P8084	*P. infestans* RC1-10	*P. infestans* T30-4
***MAKER 2 annotation***			
**Total genes**	23 457	15 893	17 475
**Genes annotated via InterPro, GO, Pfam, and SUPERFAMILY terms.**	13 659	8771	9545
**Genes annotated uniquely via BLAST against UniRef90**	1270	909	961
**Genes matching uncharacterized proteins in UniRef90**	8001	5789	6553
**Genes without assigned functional annotation**	527	424	416
**Cummulative gene coverage (bp)**	34 360 829	22 155 935	25 324 938
**Percentage of the genome covered by genes**	12.68 %	10.89 %	11.08 %
***Proteins with CAZymes functional annotation***			
**Glycoside Hydrolase Family**	327	220	217
**Glycosyltransferase Family**	187	115	124
**Polysaccharide Lyase Family**	54	30	36
**Carbohydrate Esterases**	67	38	32
**Carbohydrate-Binding Module Family**	77	41	51
**Auxiliary Activities**	48	34	32

Genes without assigned functional annotation: Those without matches to either Uniref90 or InterPro databases. Genes annotated via InterPro, GO, Pfam, and SUPERFAMILY terms: Counted as the number of gene elements in the GFF file with entries in the Dbxref field. Genes annotated uniquely via BLAST against UniRef90: Genes with functional terms derived from BLAST matches but without domain-related information from InterPro. Genes matching uncharacterized proteins in UniRef90: Genes with BLAST match to proteins labeled as uncharacterized that do not have associated functional domains. Cummulative gene coverage (bp): Cummulative number of bases from gene elements in the GFF file

[This table should appear in the same page than the Results section named *Gene prediction and functional annotation*]

A search for carbohydrate-active enzymes (CAZymes) revealed 760 proteins in *P. betacei* P8084, 478 proteins in *P. infestans* RC1-10, and 492 proteins in *P. infestans* T30-4. The majority of the recovered proteins belonged to Glycoside Hydrolases (~ 45 % of all CAZymes in the three genomes) and Glycosyltransferases (~ 25 % of all CAZymes in the three genomes). The proportions of each category were highly similar across assemblies (Table 2). Based on these results, a trophic classification was carried out to quickly assess whether differences exist in CAZymes profiles among the three assemblies. All three assemblies were classified as monomertrophs (pathogens that consume mainly simple sugars) according to their highest relative centroid distance (RCD) score for each of the three nomenclatures proposed in CATAStrophy [19] (Supplementary Table 1). This trophic phenotype is compatible with the classic biotroph category of plant pathogens.

We then counted the number of virulence-associated genes in the genome annotation files from the three assemblies. This was carried out as a naïve approach to estimate differences in genes relevant for plant-pathogen interaction. In general terms, the count of genes in the eight evaluated categories was similar for all three genomes. Our preliminary analysis showed that *Phytophthora betacei* P8084 generally has a larger number of genes per category relative to both *P. infestans* strains (Supplementary Table 2). *Phytophthora betacei* P8084 had almost twice the number of RxLR effectors (201) relative to *P. infestans* RC1-10 and *P. infestans* T30-4 (107). Crinkler (CRN) effectors were the second most abundant virulence-related genes in *P. betacei* P8084, however *P. infestans* T30-4 had the highest number of genes for the category broadly surpassing the other two assemblies. Surprisingly, *P. infestans* RC1-10 had the lowest number of CRNs of the three assemblies, breaking the similarity pattern with *P. infestans* T30-4 observed for the other categories (Supplementary Table 2).

### Orthologous genes analyses

**Fig. 2:**
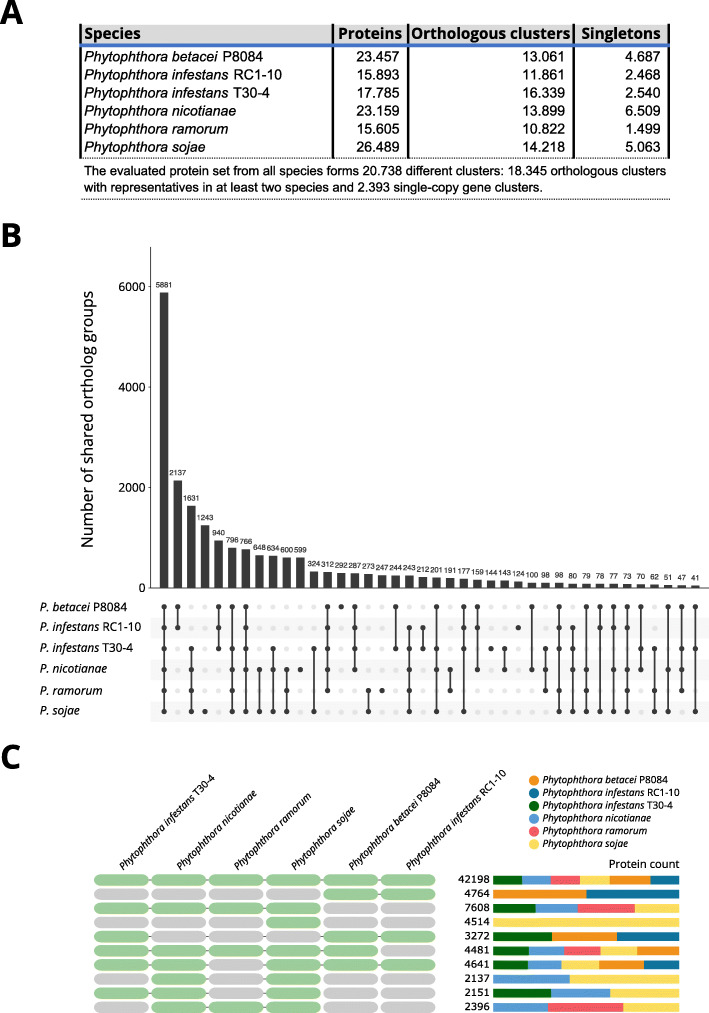
Ortholog analysis for *Phytophthora betacei* P8084, *Phytophthora infestans* RC1-10, and reference assemblies of the genus. **(A)** Summary statistics of the input dataset. This table includes the number of single proteins that did not group with any other in the dataset (singletons). (**B)** UpSet Diagram showing the number of shared ortholog clusters among the six genomes compared. (**C)** Total number of proteins and their relative proportions in the top 10 combinations by shared ortholog clusters

In order to provide a comparative context to the gene annotation of the novel assemblies, an analysis of orthologous families was carried out among the predicted proteomes of our genomes and those from the assemblies of *P. nicotianae, P. sojae, P. ramorum*, and *P. infestans* T30-4 since those are some of the most complete genomes of the genus so far. The 122 388 proteins in the dataset were grouped in 20 738 clusters, of which 18 345 were orthologous clusters that contained proteins from at least two species, and 2393 were single-copy gene clusters (Fig. 2 A). The core genome of the analyzed genomes is composed of 5881 clusters of orthologs containing 42 198 proteins from all species. This is the largest overlap partition observed in the analysis (Fig. 2B-C). Interestingly, proteins from *P. betacei* P8084 had a larger relative abundance (21.99 %) in the core genome relative to those belonging to any of the other genomes (~ 15 % each). A total of 3806 (64.7 %) of the core genome clusters had associated Gene Ontology (GO) terms. These terms are related to housekeeping functions such as cellular metabolic process (GO:0044237), nitrogen compound metabolic process (GO:0006807), RNA metabolic process (GO:0016070), and response to stimulus (GO:0050896) among many others (Supplementary Table 3).

The 2137 clusters that were exclusive to *P. betacei* P8084 and *P. infestans* RC1-10, the genomes generated with long-read sequencing, comprised the second most numerous overlap partition (Fig. 2B-C). The vast majority of the clusters of this partition (2003 clusters, 93.7 % of the total) did not have associated GO terms or any annotation at all. Those clusters that had associated GO terms, had functions similar to those from the core genome list, with housekeeping functions such as metabolism of carbohydrates and other macromolecules being the most abundant (Supplementary Tables 3 and 4). A total of 72.1 % of the genes belonging to this partition can be found inside regions annotated as transposons, including but not limited to the transposases, (1415 out of 2389 proteins from *P. betacei* P8084 and 2020 from 2375 proteins from *P. infestans* RC1-10) whereas the remaining 27.9 % were genes found outside TE- or SSR-rich areas (Supplementary Fig. 2).

A total of 1631 clusters were shared exclusively among all RefSeq genomes which were sequenced with short reads or Sanger sequencing: *Phytophthora infestans* T30-4, *Phytophthora nicotianae*, *Phytophthora ramorum*, *Phytophthora sojae*. A set of 1726 proteins from *P. infestans* T30-4 belonging to these clusters were selected for a tblastn analysis against the assemblies of *P. betacei* P8084 and *P. infestans* RC1-10 to test whether or not there was evidence of their presence in those assemblies. For *P. betacei* P8084, tblastn matches were recovered for 1714 proteins (99.3 %) of which 1683 proteins showed an H_i_ value (average between the identity and coverage percentages of the alignment) of 60 % or greater. In *P. infestans* RC1-10, 1706 proteins (98.8 %) showed matches against the assembly, of which 1669 proteins showed a H_i_ value of 60 % or greater (Supplementary Table 5).

A total of 292 orthologous clusters containing 688 proteins were exclusive to *P. betacei* P8084 (Fig. 2B-C). Of those clusters, only 27 (9.24 %) had associated functional terms, mostly with housekeeping functions. Among the GO terms recovered for these clusters, it is possible to find ATP binding (GO:0005524), DNA binding (GO:0003677), sodium ion transport (GO:0006814), L-idonate catabolic process (GO:0046183), carbohydrate:proton symporter activity (GO:0005351), motile cilium (GO:0031514), and protection from non-homologous end joining at telomere (GO:0031848), among others (Supplementary Table 6).

Finally, 124 clusters containing 282 proteins were exclusive to *P. infestans* RC1-10 (Fig. 2B-C), and only 6 clusters (4.83 % of the total, namely cluster12486, cluster18538, cluster18539, cluster18587, cluster18612, and cluster18616) had associated functional terms. These terms were related to metabolism and development: protein-lysine N-methyltransferase activity (GO:0016279), zinc ion binding (GO:0008270), long-chain fatty acid-CoA ligase activity (GO:0004467), multicellular organism development (GO:0007275), response to bacterium (GO:0009617), and hydrolase activity (GO:0016787) (Supplementary Table 7).

### Phylogeny with single-copy orthologs

**Fig. 3:**
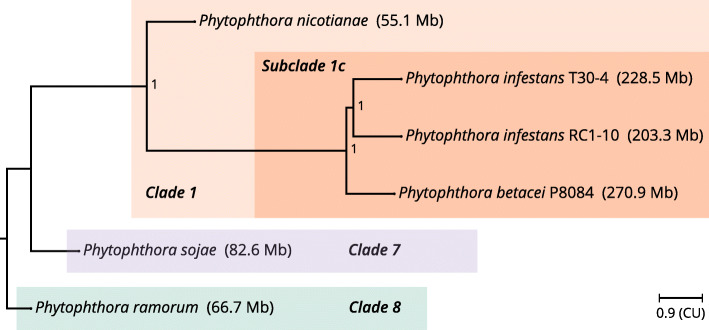
Phylogenetic placement of *Phytophthora betacei* P8084 and *Phytophthora infestans* RC1-10. A coalescent-based species phylogenetic tree was inferred using 2392 maximum-likelihood gene trees derived from single-copy orthologs shared among *Phytophthora betacei* P8084, *P. infestans* RC1-10, and other reference assemblies of the genus *Phytophthora.* The tree was rooted following the topology proposed by Yang et al. [2] and the clade nomenclature was taken from the same study. The scale is in coalescent units (CU), the numbers in the nodes indicate the local posterior probability of the branches, and the assembly size is indicated inside parentheses to the right of the name of the assembly

The phylogenetic placement of *P. betacei* P8084 and *P. infestans* RC1-10 within the genus *Phytophthora* was investigated to provide an evolutionary context for these novel genomes. We selected the 2393 single-copy orthologous clusters shared among the six analyzed assemblies as phylogenetic markers (Supplementary Table 8). The rooted coalescent-based species tree, inferred from the maximum-likelihood gene trees of each cluster, showed that both *P. infestans* assemblies form a monophyletic group that shares a relatively recent common ancestor with *P. betacei* P8084. The group *P. infestans - P. betacei* share a common ancestor with *P. nicotianae* (syn. *P. parasitica*) although the branch length of the former is greater than the latter. *Phytophthora sojae* appears as the sister group of *P. nicotianae - P. infestans - P. betacei*, and *P. ramorum* serves as the outgroup relative to all other assemblies (Fig. 3). It is noticeable that the internal branches of the group *P. nicotianae - P. infestans - P. betacei* have maximal local posterior probability support.

### Whole-genome duplication signals and synteny

**Fig. 4:**
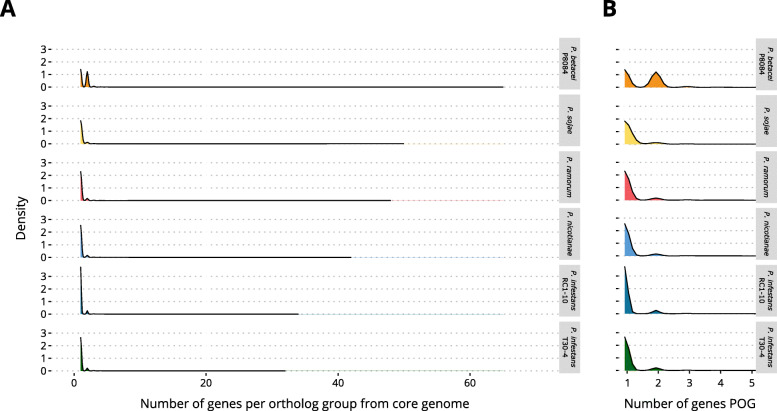
Genes per orthologous cluster (POC) from the core genome of the analyzed *Phytophthora* assemblies. (**A**) Density plot showing the distribution of genes POC for each genome included in the analysis. Since the distributions display a long tail, but most of the density is concentrated around 1 (i.e., most core ortholog clusters are composed by single copy genes), a detail of the region (**B**) is shown

To further explore signatures of evolutionary events leading to the increased genome size and number of genes of *P. betacei* P8084 relative to *P. infestans* and other species, the distribution of the number of genes per ortholog cluster from the core genome was calculated. It was observed that the assemblies with the largest number of genes: *P. betacei* P8084 (23 457) and *P. sojae* (26 489) (see Fig. 2 A for reference) contain a very large number of genes for certain core orthologous clusters relative to the rest of the compared species (Fig. 4 A). Specifically, *P. betacei* P8084 had 65 genes in a single core orthologous cluster which is the maximum value for all the genomes analyzed (Supplementary Table 9). In comparison to both assemblies of *P. infestans, P. betacei* P8084 had a few more clusters in the range of the > = 50 paralogs (Fig. 4 A). Also, it is very important to notice that *P. betacei* P8084 showed an increase in the number of core orthologous clusters with two genes relative to all other genomes analyzed (Fig. 4B), even after haplotig curation.

**Fig. 5:**
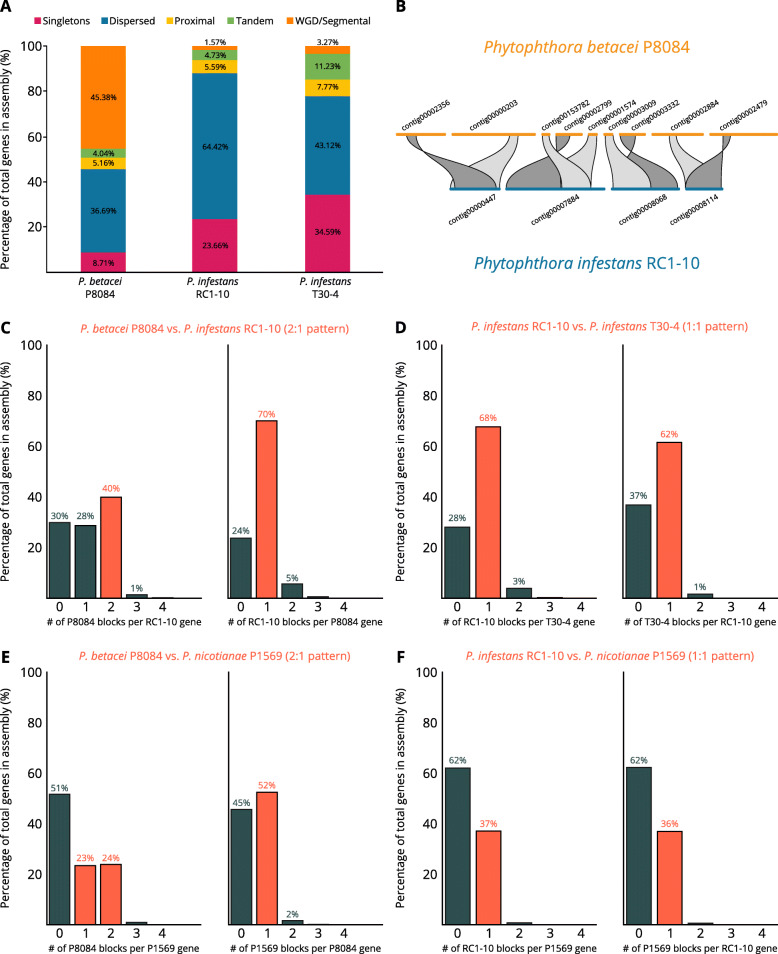
Whole genome duplication in *Phytophthora betacei* P8084 and comparative synteny analyses.** (A)** Gene duplication patterns in the assemblies of *P. betacei* P8084, *P. infestans* RC1-10, and *P. infestans* T30-4 shown as the percentage of genes classified in a given duplication type relative to all the predicted genes. (**B)** Examples of duplicated genomic regions in *P. betacei* P8084 with one single corresponding region in *P. infestans* RC1-10. Histograms showing the distribution of the number of aligned collinear anchor blocks per gene in reciprocal comparisons between: (**C**) *P. betacei* P8084 and *P. infestans* RC1-10, (**D**) *P. infestans* RC1-10 and *P. infestans* T30-4, (**E**) *P. betacei* P8084 and *P. nicotianae* P1569, and (**F**) *P. infestans* RC1-10 and *P. nicotianae* P1569. Bars in orange represent the mode of the distribution for values > 0 blocks per gene

In order to investigate whether the duplication pattern observed in the core-genome orthologs is a generalized phenomenon, we performed a duplication and collinearity analysis with all the predicted genes for the novel assemblies and compared them to *P. infestans* T30-4. The genes were classified into the following categories according to their duplication status within the genome: single-copy genes (singletons), dispersed duplicates, proximal duplicates (less than 20 genes apart), tandem duplicates (tandem), and segmental duplications. In *P. betacei* P8084, about half of the genes were contained in duplicated collinear blocks consistent with segmental duplications, and more than a third of the total genes were dispersed duplicates. Around 65 % of the genes in *P. infestans* RC1-10 were dispersed duplicates and almost a quarter of the total genes are singletons. Fewer than 2 % of the genes corresponded to segmental duplications. When comparing these two genomes to *P. infestans* T30-4, it was observed that the proportions of the categories were somewhat conserved among both *P. infestans* assemblies (showing an increase in singletons and tandem repeats in *P. infestans* T30-4) with differences attributable to sequencing effects but differed greatly from those of *P. betacei* (Fig. 5 A). A pairwise comparison among *P. betacei* P8084 and *P. infestans* RC1-10, revealed that for 300 contigs from the latter assembly there was a median duplication depth > 1, indicating that for each gene contained in those contigs there is more than one copy in *P. betacei* P8084. In some cases, complete contigs from *P. infestans* RC1-10 are contained in more than one contig from *P. betacei* P8084 (Fig. 5B). This further supports the idea that the genome size of *P. betacei* P8084 can be explained due to large-scale segmental duplication.

Reciprocal pairwise comparisons of aligned CDS between genomes showed there is further evidence of WGD in *P. betacei* P8084 relative to *P. infestans.* 40 % of the genes from *P. infestans* RC1-10 aligned to 2 collinear anchor blocks from *P. betacei* P8084 genes, compared to only 5 % in the reciprocal comparison (Fig. 5 C). Most of the genes in *P. betacei* P8084 (70 %) aligned to only one block from *P. infestans* RC1-10 genes. The same pattern was observed in a pairwise comparison among *P. betacei* P8084 and *P. infestans* T30-4 (data not shown). This evidence shows a pattern of gene duplication (2:1) in *P. betacei* P8084 relative to both *P. infestans* assemblies. A comparison between *P. infestans* RC1-10 and *P. infestans* T30-4 revealed that no whole genome duplication patterns exist between these two assemblies, since the mode of both distributions of aligned genes is 1 block per gene (1:1 pattern). It is also possible to observe that more unmatched genes exist from *P. infestans* RC1-10 when comparing to *P. infestans* T30-4 than the reverse case, possibly reflecting missing regions in the novel assembly (Fig. 5D).

**Fig. 6:**
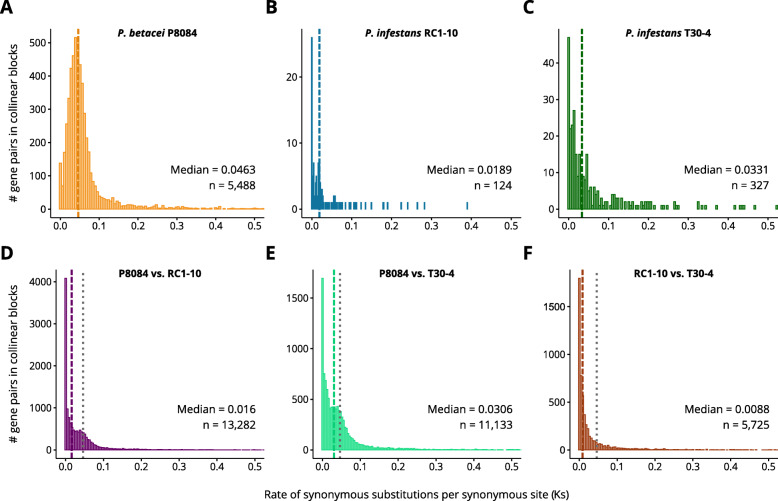
Synonymous substitutions per synonymous site rate in syntenic blocks from *Phytophthora betacei* and *P. infestans.* The distribution of Ks was obtained for pairs of genes located in syntenic collinear blocks obtained by MCScanX from alignments within and between genomes. Ks distributions of within-genome comparisons of **(A) ***P. betacei* P8084 syntenic blocks, **(B) ***P. infestans* RC1-10 syntenic blocks, and **(C) ***P. infestans* T30-4 syntenic blocks. Ks distributions of between-genomes comparisons among **(D) ***P. betacei* P8084 - *P. infestans* RC1-10 syntenic blocks, **(E) ***P. betacei* P8084 - *P. infestans* T30-4 syntenic blocks, and **(F) ***P. infestans* RC1-10 - *P. infestans* T30-4 syntenic blocks. The n represents the number of gene pairs obtained for each comparison. Dashed vertical lines represent the median of the distribution. Dotted vertical lines represent the median of the Ks distribution of *P. betacei* P8084 blocks represented in **A** for comparative purposes

In Fig. 3 it is very noticeable that a genome-size gap exists between the assemblies of the Subclade 1c and all the others. To assess if there is a genome duplication event that precedes the divergence between *P. infestans* and *P. betacei*, pairwise comparisons of collinear shared blocks were made between our two novel assemblies and *P. nicotianae* (syn. *P. parasitica*). Although there is a large fraction of the genes in both assemblies that do not share collinear anchor blocks with *P. nicotianae* and *vice versa*, it can be observed that there are signals of WGD in *P. betacei* P8084 relative to *P. nicotianae* (Fig. 5E) but a 1:1 pattern is observed between *P. infestans* RC1-10 and *P. nicotianae* (Fig. 5 F). Additional evidence to support this claim comes from an analysis of rate of synonymous substitutions per synonymous site (Ks) in syntenic collinear gene blocks. In comparisons within the same genome, *P. betacei* P8084 shows a pronounced peak resembling a gaussian bell centered around 0.046 (Fig. 6 A). In contrast, the Ks distributions of both *P. infestans* highly resemble an exponential distribution, with median values lower than *P. betacei* P8084. The difference of collinear gene pairs between the two species is noticeable (Fig. 6B-C). The comparison between *P. betacei* P8084 - *P. infestans* RC1-10 and *P. betacei* P8084 - *P. infestans* T30-4 revealed bimodal Ks distributions. In both cases, the tallest peak is centered around Ks = 0 and the secondary peak is centered around 0.03 (Fig. 6D-E) which is lower than the median of the distribution of *P. betacei* P8084. The comparison between assemblies of *P. infestans* showed a distribution similar to an exponential one, which is expected for species without WGD (Fig. 6 F). Taken together, all this suggests that *P. betacei* P8084 underwent a lineage-specific WGD process that occurred after the divergence with *P. infestans*.

### Transposable elements and short-sequence repeat annotation

In order to investigate the diversity of transposable and repetitive elements in the genome, the catalog of non-redundant transposable elements (TE) and short-sequence repeats (SSR) of *P. betacei* P8084 and *P. infestans* RC1-10 was inferred. For comparison purposes, we also included *P. infestans* T30-4 in the analysis.

**Fig. 7:**
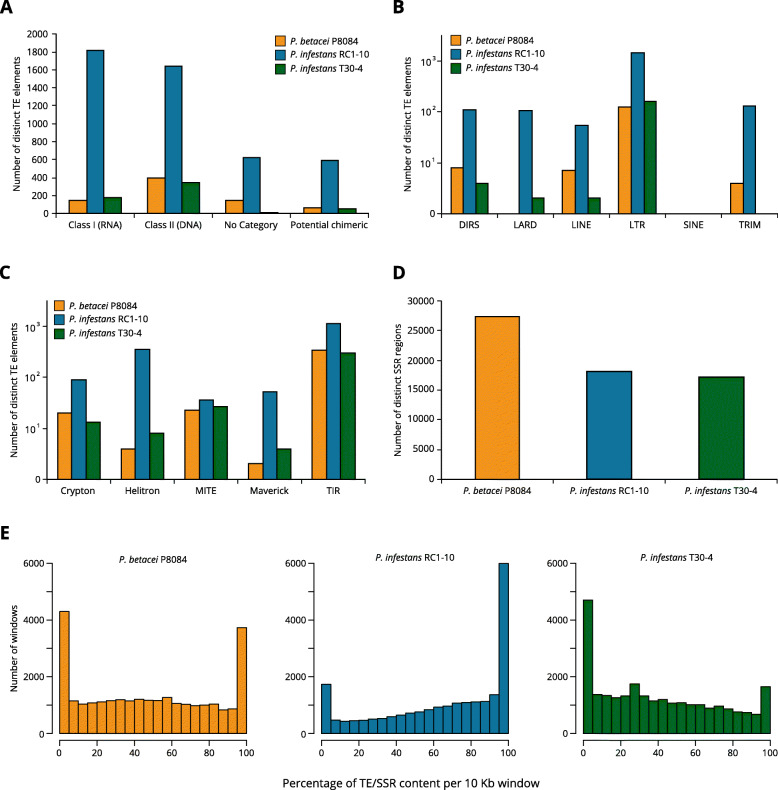
Non-redundant catalog of transposable and repetitive elements from *Phytophthora betacei* P8084 and *P. infestans* assemblies.** (A)** Number of distinct transposable elements per class in the catalog. (**B)** Distinct Class I (RNA transposons or retrotransposons) elements by order. (**C)** Distinct Class II (DNA transposons) elements by order. (**D)** Number of distinct short-sequence repeats (SSRs) in the assemblies. (**E)** Distribution of annotated transposable elements (TEs) and short-sequence repeats (SSRs) in the assemblies of *P. betacei* P8084, *P. infestans* RC1-10, and *P. infestans* T30-4. The data is presented as the percentage of bases annotated as TEs or SSRs per 10 Kb windows for each genome

Six different orders of retrotransposons (Class I transposons) and five orders of DNA transposons (Class II transposons) could be recovered for all the genomes (Fig. 7). Before filtering potential chimeric elements and potential host genes, Class I transposons comprised 19.6 %, 43 %, and 32.7 % of the total diversity of distinct TEs identified for *P. betacei* P8084, *P. infestans* RC1-10, and *P. infestans* T30-4 respectively (Supplementary Table 10). Class II transposons represented 52.4 %, 38.9 %, and 62.9 % of the total diversity of TEs in *P. betacei* P8084, *P. infestans* RC1-10, and *P. infestans* T30-4, respectively (Supplementary Table 10).

Unclassified repetitive elements (i.e., those that could not be assigned to a particular category) represent 19.3 % and 14.8 % of the total diversity of TEs in *P. betacei* P8084 and *P. infestans* RC1-10, respectively. This contrasts with *P. infestans* T30-4 where only 0.5 % of the distinct TEs could not be classified (Fig. 7 A, Supplementary Table 10).

For all TE classes examined in the non-redundant catalog, including uncategorized and potential chimeric elements, *P. infestans* RC1-10 consistently showed a greater number of unique transposon types than *P. betacei* P8084 and *P. infestans* T30-4, up to two additional orders of magnitude in certain TE taxonomic orders, despite its smaller genome size (Fig. 7 A). The Long Terminal Repeats (LTR) order of retrotransposons appeared to be the most diverse type of Class I transposons in the three species. For the other main orders of retrotransposons, the number of elements was also higher for *P. infestans* RC1-10 relative to *P. betacei* P8084 and *P. infestans* T30-4. This was particularly noticeable in the case of Large Retrotransposon Derivatives (LARDs) (Fig. 7B). In the case of Short Interspersed Nuclear Elements (SINEs) there was only one type of element for *P. betacei* P8084 and *P. infestans* RC1-10, whereas no SINEs could be found in *P. infestans* T30-4 (Fig. 7B). In Class II transposons, the Terminal Inverted Repeats (TIR) order was the most diverse in all three genomes, although the number of unique TIRs was greater in *P. infestans* RC1-10 relative to the other two assemblies (Fig. 7 C). The three genomes shared a similar degree of diversity for Miniature Inverted-repeat Transposable Elements (MITEs) order. Helitrons were the second most diverse order in *P. infestans* RC1-10 followed by Cryptons and Mavericks. The second most diverse order of TEs in *P. betacei* P8084 was MITE followed by Cryptons. A very similar pattern was seen in *P. infestans* T30-4 (Fig. 7 C). When looking at the number of SSRs across the three genomes, *P. betacei* P8084 was the species with the greatest number of simple repeats relative to both assemblies of *P. infestans* which showed similar values (Fig. 7D). It is interesting to note that a greater diversity of TEs was recovered from *P. infestans* RC1-10 than from *P. infestans* T30-4.

Non-redundant repetitive element catalogs only describe the diversity of the elements found in a genome, but do not explain the contribution of such elements to the genome size of an organism in terms of their copy numbers or their completeness. After matching the TE and SSR catalog to each genome to find the number of copies of each element, it was found that TEs and SSRs comprised 47.31 % of the *P. betacei* P8084 genome, 68.85 % of the *P. infestans* RC1-10 genome, and 44.20 % of the *P. infestans* T30-4 genome. These numbers represent 128 Mb, 141 Mb, and 102 Mb of the respective assemblies. In the case of SSRs, a total of 24 967 repeats were found for *P. betacei* P8084, 16 224 were found for *P. infestans* RC1-10, and 15 563 were found for *P. infestans* T30-4. A total of 2312 low complexity regions were annotated in *P. betacei* P8084 compared to 1806 in *P. infestans* RC1-10, and 1609 in *P. infestans* T30-4 (Table 3, Supplementary Table 10).

**Table 3:** Annotation statistics of transposable elements (TEs) of *Phytophthora betacei* P8084 and *P. infestans* RC1-10 and T30-4 assemblies

	*P. betacei* P8084	*P. infestans* RC1-10	*P. infestans* T30-4
**Cumulative TE coverage (bp)**	128 165 366	141 368 243	102 711 821
**Percentage of the genome covered by TE (%)**	47.31	68.85	44.20
**Total number of TE fragments**	75 950	85 415	65 054
**Total number of full-length fragments**	12 279 (16.17 %)	11 954 (14.00 %)	6314 (9.71 %)
**Total number of TE copies**	63 842	65 956	55 488
**Total number of full-length copies**	13 245 (20.75 %)	12 961 (19.65 %)	6731 (12.13 %)
**Total number of simple repeats (SSRs) and low complexity regions**	27 325	18 019	17 172
**Total combined cummulative coverage of simple repeats (SSRs) and low complexity regions (bp)**	1 089 595	744 317	764 909

[This table should appear in the same page than the Results section named *Transposable elements and short-sequence repeat annotation*]

We calculated the percentage of bases belonging to repetitive elements in 10 kb non-overlapping windows across the novel assemblies as a first approach to the contribution of such elements to the genome size (Fig. 7E). Most of the windows examined in the three genomes either come entirely from repetitive elements or from regions that do not contain any (the two extremes of the distribution). This can be observed in the histograms of *P. betacei* P8084 and *P. infestans* RC1-10, as the two largest peaks in both distributions correspond to windows with 0 % and 100 % repetitive content. In contrast, in the histogram of *P. infestans* T30-4 the second largest peak is located near 30 % and it is marginally larger than the peak at 100 %. A higher number of windows that do not come from TEs or SSRs was observed in *P. infestans* T30-4 and *P. betacei* P8084 relative to *P. infestans* RC1-10. Consistently, a lower number of windows in the first two genomes were part of repetitive elements, being this count notably lower in *P. infestans* T30-4. Further annotation details can be found in Table 3.

### Gene-dense and gene-sparse genomic architecture analysis

It has been previously reported for other species of genus *Phytophthora* that their genomes have an architecture characterized by clusters of essential genes for the organism (gene-dense regions, GDRs) separated by highly repetitive regions filled with TEs and SSRs (gene-sparse regions, GSRs) [8]. In order to investigate the genomic architecture in *P. betacei* P8084 and *P. infestans* RC1-10, we measured the 5’ and 3’ distances between genes, also named flanking intergenic regions (FIRs). Genes that were located inside TEs were excluded from the analysis. Using the FIRs distances, we estimated a genomic distance cut-off such that for a certain gene, if the distance to its closest gene is lower than the cut-off it can be said that it is located within a gene-dense region. On the contrary, if the closest gene is further apart than the cut-off, it is located in a gene-sparse region. In-between regions are the border areas where on one side the distance to the next gene is lower than the cut-off, and on the other side it is greater.

Since core orthologs are expected to be enriched in the GDRs, the selection criterion to define the cut-off value (L) was the number of core orthologs located in the putative gene-dense regions of each genome. For *P. betacei* P8084 the calculated cutoff value was L = 4700 bp, and for *P. infestans* RC1-10 was L = 7200 bp. These were the values that maximized the segregation rate of core orthologs given that the percentage of such genes in GSR or in-between regions did not grow noticeably at higher values of L (reached saturation). See Supplementary Fig. 3 for more details.

**Fig. 8:**
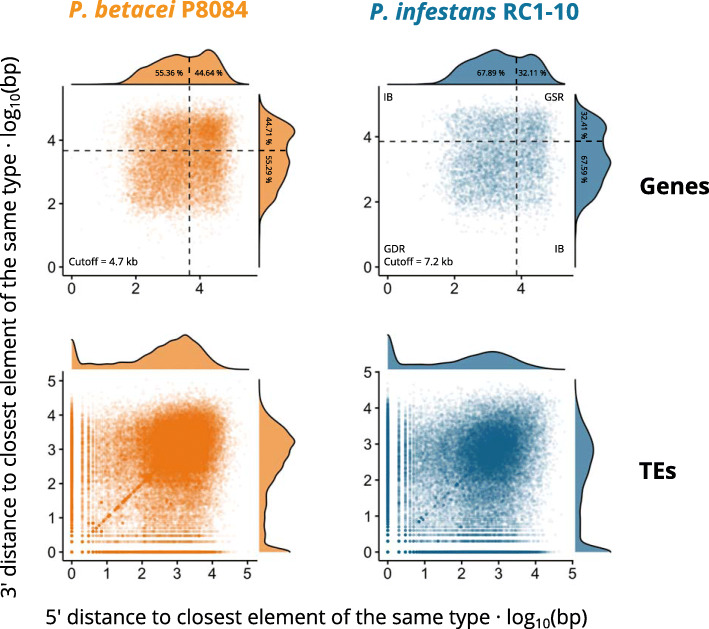
Genome architecture analysis of *Phytophthora betacei* P8084 and *P. infestans* RC1-10. Bivariate distributions of the 5’ and 3’ distances of genes and transposable elements (TEs) to the closest elements of the same type for *P. betacei* P8084 and *P. infestans* RC1-10. Dashed lines represent the estimated flanking intergenic region (FIRs) distance cutoff L used to define gene-dense regions (GDRs), gene-sparse regions (GSRs), and in-between (IB) regions in the assemblies whenever possible. The numbers inside the density plots represent the proportion of the total density of the distribution below and above the value L

In Fig. 8 a bimodal distribution can be observed for FIRs in both genomes, revealing groupings of genes at distances ~ 10^3^ bp and another peak at ~ 10^5^ showing more separated genes. In *P. betacei* P8084 the highest peak is the one located at ~ 10^5^ bp whereas in *P. infestans* RC1-10 the highest one is located around ~ 10^3^ bp. The calculated cutoff value for each genome points to a valley between the two modes.

The 3’ and 5’ distance measurements were also carried out for the TEs (Fig. 8). In *P. betacei* P8084 it is possible to observe a high peak of distances around ~ 10^3^ bp and a peak near ~ 10^0^ bp. In *P. infestans* RC1-10 a large peak is observed around ~ 10^0^ bp with a small peak around ~ 10^3^ bp. Peaks around 10^0^ bp are evidence of tightly clustered TEs in both genomes, being much more frequent in *P. infestans* RC1-10. The peak around 10^3^ bp in *P. betacei* P8084 shows that TEs are more spaced between them relative to *P. infestans* RC1-10.

## Discussion

The objective of our study was to sequence and to annotate the related genomes of *Phytophthora betacei* P8084 and *P. infestans* RC1-10 to understand their pattern of genome divergence and evolution. *Phytophthora betacei* P8084 is an oomycete plant pathogen with high host preference for *Solanum betaceum* in natural settings while *P. infestans* RC1-10 was isolated originally from potato and it is believed to have a higher host range. Although these two species are related and belong to the same subclade (clade 1c, which is part of the broader Clade 1, Fig. 3) of the genus *Phytophthora*, limited gene flow is estimated to occur between them, as evidenced in the original species description [5].

Here, our results showed that *P. betacei* has the largest sequenced genome size of the *Phytophthora* genus so far with 270 Mb. The large genome of *P. betacei* is probably due to an independent species-specific whole genome duplication, whereas *P. infestans* RC1-10 has expanded its genome (203 Mb) relative to the ancestor of the Clade 1 under the activity of transposable elements in the same way described for *P. infestans* T30-4 [12]. However, similar results could be observed from a hybridization between two closely related, yet undescribed, species, but experiments and analysis to discriminate between these possibilities are beyond the scope of the current manuscript.

Several mechanisms contribute to genome size variation in eukaryotes from yeast to more complex genomes like vertebrates. Two of the most important ones are whole genome duplications, due to either autopolyploidy or hybridization, and the accumulation of transposable elements. Whole genome duplications have been extensively studied in plants since it was discovered in the *Arabidopsis* sequenced genome back in 2000 [20], and it has been reported for oomycetes in a recently published study on *P. megakarya* and *P. palmivora* [18]. It is interesting to note that in some cases polyploid genomes are able to return to disomy through a diploidization process such as gene loss, mutation and sub-functionalization, among other mechanisms [21]. These processes have deep consequences on the fate of the paralogous gene copies. WGD is thought to participate in the evolution and adaptation of organisms [22], notably in plants where several studies and methodologies have been developed to detect and understand the relevance of paleo-polyploids in genomic sequences [23].

Our analysis strongly suggests a lineage-specific whole genome duplication in *P. betacei.* We are aware that the large size of the *P. betacei* genome relative to other assemblies in the genus is not in itself a strong criterion to consider the presence of a WGD. However, several lines of evidence suggest that a WGD indeed occurred in *P. betacei.* First, the presence of duplicated genes in the BUSCO analysis, the distribution of genes per core ortholog group across assemblies, and the larger fraction of proteins from *P. betacei* in the core genome led us to consider WGD. This scenario is fully supported by the presence of duplicated blocks in the synteny analysis with *P. infestans* and the Ks curve distribution with a single peak. On the other hand, both *Phytophthora infestans* assemblies analyzed in this study exhibit genome sizes greater than 200 Mb, much larger than those from *P. nicotianae*, *P. sojae*, and *P. ramorum*, but no WGD event was detected in those assemblies. *Phytophthora infestans* RC1-10 did not show any signal of whole-genome duplication, but extensive dispersed duplications (i.e., non-adjacent duplicated genes) and some short tandem duplicates were observed. A similar duplication pattern was observed for *P. infestans* T30-4, although a smaller proportion of dispersed duplications could be identified in such assembly.

Whole genome duplication signals were previously detected in the *Phytophthora* genus, suggesting that the process has shaped the evolution of several species or even the entire genus. Martens and coworkers predicted the presence of hidden duplications in the ancestor of *P. infestans, P. sojae and P. ramorum* [24] in the form of multiple small blocks of duplicated genes. Such small blocks could suggest extensive rearrangements since the duplication process in the ancestors of the *Phytophthora* species. However, in a comparative study of small blocks consisting of two directly adjacent genes in *Phytophthora*, it was postulated that the origin of that kind of duplications is likely to be lineage-specific duplication bursts due to TE activity rather than WGD events [13]. More recently, independent whole genome duplications have been detected in the genomes of *P. megakarya* and *P. palmivora*, two closely related species in the *Phytophthora* clade 4, with deep consequences on the expansion of gene families, including RxLR effectors [18].

Similarly to *P. megakarya* and *P. palmivora*, only one peak was observed in the Ks curve distribution of *P. betacei* suggesting that the cause of the duplication could be an autopolyploidy event rather than allopolyploidy [25]. This WGD event occurred independently from those in *P. megakarya* and *P. palmivora* in the clade 4, evidencing that WGD is more frequent than previously thought in the *Phytophthora* genus. Furthermore, the WGD occurred specifically in *P. betacei* but not in *P. infestans* suggesting a relatively recent event. Although we did not date the WGD in *P. betacei*, the median value of the Ks distribution in *P. betacei* (0.046 Ks) is similar to the peaks from *P. megakarya* (0.021 Ks) and *P. palmivora* (0.040 Ks). In those two species, duplication was estimated at 0.88 and 1.53 My, respectively, long after their estimated date of divergence (16.4 My) and the estimated divergence for the genus (26.6 My) [26].

Although *P. infestans* does not appear to have undergone whole genome duplications, its genome size is large and relatively close to that of *P. betacei* (203 Mb in *P. infestans* RC1-10 versus 270 Mb). The origin of this genomic content is found in a high diversity and abundance (in terms of copy number) of transposable elements. These results fit with the current models for genomic expansion in filamentous plant-pathogens that propose the accumulation of TEs and other repetitive sequences as one of the main causes [7]. A large difference in the TE content between *P. betacei* P8084 and *P. infestans* RC1-10 is evident, mainly for all categories of retrotransposon families and Helitrons. Long Terminal Repeat retrotransposons transpose via a duplicative mechanism (using an RNA intermediate) that allows them to reach high copy numbers in a genome and are well known to participate in the genome size variation of plants and animals [27]. In rice, the massive amplification of only 3 families of LTR retrotransposons resulted in the doubling of the genome size in a few million years [28]. In conifers, the number of copies of Ty3/Gypsy and Ty1/Copia LTR retrotransposons accumulated slowly over tens or hundreds of millions of years leading to an increase in the genome sizes of at least 5 species [29].

Differences in the TE content were also observed at the intraspecific level between *P. infestans* RC1-10 and *P. infestans* T30-4. We propose that long-read sequencing technologies allow the access to repetitive elements that previously could not be easily recovered with short-read technologies, most probably due to mis-assembly and assembly collapse. In the latter, the copy number of repetitive transposons in a stretch of DNA is underestimated due to algorithmic limitations of the assemblers [30]. An argument for this comes from the fact that most of the ortholog families found exclusively in the long-read assemblies generated in this study were unclassified genes contained in transposons. Also, an additional hint comes from the presence of hundreds of uncategorized TEs recovered from the two novel assemblies (144 in *P. betacei* P8084 and 628 in *P. infestans* RC1-10), in contrast with only 3 found in *P. infestans* T30-4. Additionally, in our analyses, *P. infestans* T30-4 showed a different genomic transposon content than previously reported. Our TE annotation procedure revealed that only 44.2 % of this genome was composed by TEs, which contradicts the 74 % in the original genome report [12]. The TE annotation strategy used for *P. infestans* T30-4 (RepeatScout) in that report was different to ours, so we attempted to replicate the TE coverage claimed by the original paper using the same tool with no success (data not shown). Taken together, long-read technologies emerge as a new opportunity for research in the field of mobile DNA. We also suggest that there is a need for revision of the TE annotation of the *P. infestans* RefSeq genome.

In the current version of the manuscript, we present a preliminary evaluation of virulence factors, however, in parallel, a thorough analysis was performed on a draft version of our genome by Rojas-Estévez et al. [31]. We thus reference to that manuscript to anyone interested in more detailed analysis of the virulence factors. Nonetheless, we find value in the fact that the number of genes per category of these virulence-related genes was consistent among the *P. infestans* assemblies for the most part. Taking this together with the BUSCO completeness assessments, and the similarities found in the CAZymes and CATAStrophy functional gene profiling, we consider that *P. infestans* RC1-10 assembly is a valuable genomic resource that is semantically comparable to the established *P. infestans* reference genome, and that can be used to delve deeper into the genetics of the EC-1 Andean lineage of the pathogen.

We did not find evidence to support the claim of the GDR/GSR architecture in *P. betacei* P8084. In this work, we excluded the regions in the genome annotated as TEs and SSRs to obtain a more accurate measurement of the FIRs without the bias of including transposon genes. Upon performing the FIR analysis proposed by Raffaelle et al. [8], we found out that the fraction of annotated genes from *P. betacei* P8084 within the putative GSR was not very different from the one in GDR. Also, TEs are more evenly distributed in space in *P. betacei* P8084 than in *P. infestans* RC1-10, where the GDR/GSR architecture can be observed. We then conclude that there is no evidence to support GDR/GSR architecture in *P. betacei* P8084. As an additional note, the cut-off value L used to define GSRs that we found for *P. infestans* RC1-10 (7.2 Kb) is almost 5 times greater than the one reported in Raffaelle et al. [8]. This is most probably caused, as we specified before, due to the exclusion of TEs from the analysis.

Having an annotated genome allowed us to delve into the phylogenetic relationships of *P. betacei* P8084 by using single-copy orthologs as phylogenetic markers in multispecies coalescent models [32]. In the species description of *P. betacei* by Mideros et al., [5] a SNP phylogeny grouped the candidate *P. betacei* individuals of the species together in a monophyletic clade with *P. andina* as its sister species. Using this approach, *P. infestans* emerged as the sister group of *P. betacei - P. andina*. In the same study, a second phylogeny derived from mitochondrial genomes placed *P. andina* and *P. betacei* as a derived lineage of *P. infestans*. Our results provide support for the phylogenetic hypothesis where *P. betacei* emerges as a standalone species. Phylogenomic methods that use orthologs instead of a few selected mitochondrial and nuclear markers still exhibit variability in the placement of major clades within the genus *Phytophthora* relative to the root (e.g., clades 7 and 8) [33, 34]. However, all the species in the Clade 1 show a stable position that is consistent with all phylogenies reviewed in this study and their relationships are well resolved.

Besides allowing phylogenetic inference, the analysis of orthologs (both BUSCO and OrthoVenn analyses) served as a powerful tool for assembly and annotation evaluation. First, the most important hints pointing towards a WGD event in *P. betacei* P8084 came from the BUSCO duplications and the number of proteins from this assembly in the core genome. Second, finding that one of the largest groups of shared ortholog families was the one composed by orthologs exclusive from short-read assemblies led us to think about the quality of the assembly. In our results, almost all those orthologs could be found as fragmented alignment matches with the nucleotide sequences of both *P. betacei* P8084 and *P. infestans* RC1-10. This indicates an opportunity to further improve the quality of these assemblies using complementary sequencing datasets, since these might alleviate the frequent indel errors present in PacBio and long-read sequencing technologies leading to failure in gene prediction by, for example, introducing frameshift errors [35]. Finally, as mentioned before, most of the ortholog families found exclusively in the long-read assemblies generated in this study were unclassified genes contained in transposons which bears testimony of the potential of the long-read technologies to allow the discovery of novel repetitive elements with yet-to-discover functions and implications for the genomes of organisms.

## Conclusions

Two different mechanisms of genome evolution have been identified in two closely related species from the same *Phytophthora* subclade. *Phytophthora betacei* P8084 has the largest genome of the genus due to a lineage-specific process of whole genome duplication. The genome size of *Phytophthora infestans* RC1-10 is the result of the invasion of diverse transposable elements throughout its evolutionary history. These mechanisms are some of the most important drivers of genome architecture, size, and evolution in eukaryotes. Thus, these genomes represent a unique opportunity to study the consequences of such divergent evolutionary paths in the virulence and host range specificity of these pathogens, among many other traits.

We did not find evidence to support the idea that the genome of *P. betacei* P8084 follows the same gene-dense/gense-sparse architecture proposed for *P. infestans* and other filamentous plant pathogens. One of the questions arising from this fact is whether *P. betacei* represents an exception to the “two-speed genome” hypothesis or if maybe there has not been enough evolutionary time for this genomic dynamic to consolidate. Finally, long-read sequencing is a valuable technology for the study of challenging oomycete genomes, since it allows the access to genomic elements (such as transposable elements) that could not be recovered before due to the limitations of short-read sequencing technologies.

## Materials and methods

### Isolates and sequencing

The biological material used for this study was obtained from the microorganisms collection of the Museum of Natural History of Universidad de los Andes, Colombia, under accession numbers ANDES-F 1172 (*P. betacei* P8084) and ANDES-F 1833 (*P. infestans* RC1-10). The usage of the genetic material of *Phytophthora betacei* P8084 and *P. infestans* RC1-10 isolates here studied is licensed by the Colombian government to the researchers through the “Contrato de Acceso a Recursos Genéticos y sus productos derivados, N°128, 23/09/2016, MADS-SRR”. In brief, the origin of the deposited material was for *Phytophthora betacei* P8084 from a single zoospore isolate obtained from a late blight-affected tree tomato (*Solanum betaceum)* plant in the municipality of Colón, Putumayo, Colombia as described in Mideros et al. [5]. *Phytophthora infestans* RC1-10 is a single zoospore isolate obtained from an affected potato plant in the municipality of El Rosal, Cundinamarca, Colombia.

The mycelium of each isolate was grown in Plich liquid medium [36] for 15–20 days in the dark at 20 °C, then harvested, washed, and disrupted in liquid nitrogen. DNA extraction from the disrupted mycelium samples and PacBio Sequel sequencing was carried out by Novogene Corporation (Hong Kong, China) for isolate P8084 and by BGI (Hong Kong, China) for isolate RC1-10. The raw sequencing yield was ~ 17.5 Gb for P8084 and ~ 9.2 Gb for RC1-10. Additionally, an Illumina PE 100 bp library for P8084 containing ~ 22.7 Gb sequenced by Mideros et al. [5] was used for assembly polishing and further analyses.

### Genome assembly and polishing

Long reads of both isolates were corrected and assembled using Canu v. 1.6 [37] with the default parameters except for those relative to computing resource usage (30 cores, 128 GB of RAM). The required genome size value, used in the assembler’s correction step, was set to 230 Mb for *P. infestans* RC1-10 given the length of the RefSeq assembly for the species, and to 400 Mb for *P. betacei* P8084 as an approximate value based on the previous flow cytometry genome size estimations made by Mideros et al. [5].

Two polishing rounds were performed for each assembly. For the first polishing round, the uncorrected sequencing subreads were aligned to the assembly using pbmm2 (https://github.com/PacificBiosciences/pbmm2, v. 0.9.0) wrapper for minimap2 aligner [38]. Variant calling and reference correction were performed using Arrow v. 2.2.2 from the GenomicConsensus package (Pacific Biosciences) with the option for detection of diploid heterozygous variants enabled. Two different approaches were used for the second polishing round: first, Illumina paired reads were aligned to the polished assembly of P8084 with Bowtie2 [39] and variant calling and reference correction was performed using NGSEP v. 3.3.0 [40]; second, the corrected-trimmed subreads obtained from Canu were aligned to the polished assembly of RC1-10, and variant calling and correction was also performed with NGSEP. Genome quality improvement was evaluated by single-copy ortholog analysis with BUSCO v. 3.0.2 [41] with the Stramenopila-Alveolata orthologous gene set, and several assembly statistics were assessed with QUAST v. 4.4 [42].

### Haploid representation of the genomes

Illumina PE data from *Phytophthora betacei* P8084 was analyzed with GenomeScope v. 2.0 suite using default parameters [43] to obtain an estimation of ploidy and heterozygosity. Assembly curation to obtain a haploid representation of each genome was carried out using the Purge Haplotigs pipeline [44]. Sequencing subreads were aligned to the polished assemblies with pbmm2, the coverage cutoff values to exclude high-coverage misassembled repeats and low-coverage contigs were selected manually following the package documentation. For *P. betacei* P8084 two curation rounds were carried out. In the first round three cutoff values (60 %, 70 %, and 80 %) were evaluated for their effectivity in haplotig reduction with BUSCO. For the second round the same steps were followed but the alignment cutoffs evaluated were only 70 % and 80 %. For *P. infestans* RC1-10 a single purging step was carried out with the same three alignment cutoff values mentioned above, but deactivating the coverage contig filtering option for the 60 % alignment cutoff.

### External data sources

The assemblies and gene annotations of *P. infestans* strain T30-4 (NCBI Assembly Accession: GCA_000142945.1), *P. nicotianae* (syn. *P. parasitica*) strain P1569 (NCBI Assembly Accession: GCA_000365505.1), *P. sojae* strain P6497 (NCBI Assembly Accession: GCA_000149755.2), and *P. ramorum* strain Pr102 (NCBI Assembly Accession: GCA_000149735.1) were downloaded from the NCBI Assembly repository. Additionally, *P. infestans* T30-4 transcriptome and proteome files were used as evidence for annotation tasks.

### Gene prediction and functional annotation

MAKER 2 (v. 2.31.9) [45] annotation pipeline was used to perform gene prediction and annotation of both *P. betacei* P8084 and *P. infestans* RC1-10. Within the pipeline, *ab initio* gene predictions were done using AUGUSTUS v. 3.0.3 [46] on a repeat masked genome, and further refining of gene models was done using *P. infestans* T30-4 transcriptome and proteome derived from RefSeq database (NCBI). Functional assignment of the transcripts was carried out by BLAST searches against UniRef90 database [47] and by protein domain identification using InterProScan v. 5.25-64.0 [48]. The results of these searches were integrated to the predicted gene models using the *iprscan2gff3* script included in MAKER 2. Finally, the resulting annotation was further curated by calculating the distribution of the Annotation Edit Distance (AED) values from the GFF file containing the predicted transcripts. A quality filter for the gene models was applied using the MAKER script quality_filtering.pl with an exclusion threshold of AED > = 0.3. A reannotation of *P. infestans* T30-4 RefSeq assembly was carried out with the same parameters and input data for comparison purposes.

To refine the annotation quality of proteins relevant to the pathogenesis process in plants, two brief analyses on carbohydrate-active enzymes (CAZymes) and virulence-related genes were performed in the predicted proteomes of the two novel assemblies and the RefSEq. For the CAZymes annotation, a local version of the dbCAN2 server was used to annotate the protein sequences containing domains related to carbohydrate metabolism [49]. This CAZyme analysis was used to classify the organisms into the trophic phenotypes proposed by Hane et al. [19] using the CATAStrophy software (v. 0.0.3) described in such study.

In order to perform a naïve comparison of virulence-related genes, eight categories were searched in the annotation GFF3 files from *P. betacei* P8084, *P. infestans* RC1-10, and *P. infestans* T30-4 following the classification proposed by Armitage et al. [50]: Crinkler effectors (CRNs), cutinases, elicitins, necrosis-inducing effectors (NLPs), phytotoxins, protease inhibitors, RxLR effectors, and other virulence-related proteins. The gene search was done with GNU grep command line tool using specific search terms for each category (Supplementary File 1) that included InterPro ontology terms, Pfam terms, and gene names among others.

### Analysis of orthologous genes

The suite OrthoVenn2 [51] was used to obtain clusters of orthologous genes from the novel assemblies (*P. betacei* P8084 and *P. infestans* RC1-10) and from the selected reference genomes of the genus (*P. infestans* T30-4, *P. sojae*, *P. ramorum*, and *P. nicotianae*) using their predicted protein sets as inputs. The visualization of the shared clusters among assemblies was carried out with UpSetR package [52].

To figure out whether the ortholog clusters shared exclusively among *P. betacei* P8084 and *P. infestans* RC1-10 were derived from transposons, we used BEDTools v. 2.25.0 [53] to subtract the regions annotated as transposons from the MAKER 2 annotation of each genome. Then, we counted the number of genes from the intersection between *P. betacei* P8084 and *P. infestans* RC1-10.

A tblastn analysis [54] was run against the assemblies of *P. betacei* P8084 and *P. infestans* RC1-10 using the set of orthologs of *P. infestans* T30-4 shared exclusively with the other assemblies as input. Individual alignment matches with identity lower than 60 % were filtered out. The coverage percentage of a protein for a certain contig was calculated as the number of uniquely matched positions of the protein, considering all hits in the given contig, divided by its length. The identity percentage of an aligned protein was the average of the identity of all the matches of such protein with the contig. Finally, the harmonic mean between coverage and identity percentages was calculated to obtain a value that we denominated H_i_ index. If a protein had matches in more than one contig, only the contig with the best H_i_ was selected.

### Phylogeny with single-copy orthologs

A total of 2392 clusters of single copy orthologs shared among all the evaluated assemblies were used to infer a species phylogenetic tree. The proteins of each cluster were aligned with MAFFT v. 7.471 [55] using the G-INS-i strategy. Then, the best maximum likelihood tree for each protein cluster was inferred using RAxML v. 8.2.12 [56]. For each tree, the selection of the best amino acid substitution model was done using the -PROTGAMMAAUTO feature of RAxML and a rapid Bootstrap analysis was made with 500 repetitions. After the best 2392 gene trees were obtained, the unrooted coalescent-based species tree was inferred with ASTRAL-III v. 5.7.3 [57]. The tree was rooted following the topology proposed by Yang et al. [2] by assuming a midpoint root that is the last common ancestor between *P. ramorum* (taken as an outgroup for this analysis) and all the other assemblies. *Phytophthora sojae* and *P. ramorum* were selected on the basis that they belong to the proposed monophyletic clades 7 and 8 of the genus, respectively, that diverged earlier than the proposed clade 1 of which *P. nicotianae* and *P. infestans* that are members [2].

### Whole-genome duplication and synteny analyses

We performed an all-by-all blastp [54] alignment with the proteins coded by the predicted genes within each assembly for *P. betacei* P8084, *P. infestans* RC1-10, and *P. infestans* T30-4. The best five non-self hits in each target genome that met an *E*-value threshold of 10^− 5^ were reported. MCScanX [58] was then used to discover and classify gene duplications in each genome. The compara module of the JCVI utilities (v. 1.0.9 + 6.g6732285f) [59] was used to visualize contig-level duplications in *P. betacei* P8084 relative to *P. infestans* RC1-10, and was also used to obtain histograms of aligned blocks per gene in pairwise comparisons between *P. betacei* P8084, *P. infestans* RC1-10, *P. infestans* T30-4, and *P. nicotianae* (syn. *P. parasitica*) P1569. This module uses the MCScan pipeline [60] that recovers LAST [61] alignments of the CDSs from predicted genes of each genome to find the best aligned blocks between genomes.

### Analysis of synonymous substitutions per synonymous site

The rate of synonymous substitutions per synonymous site (Ks) was calculated for each one of the duplicated gene pairs found in syntenic collinear blocks identified by MCScanX. This analysis was performed for *P. betacei* P8084, *P. infestans* RC1-10, and *P. infestans* T30-4 using the add_ka_and_ks_to_collinearity.pl script included in the MCScanX package. Plots were truncated at Ks = 2 for visualization purposes.

### Transposable and repetitive elements annotation

A curated non-redundant catalog of transposable elements (TEs) and repetitive regions in both *P. betacei* P8084 and *P. infestans* RC1-10 genomes was obtained with REPET TEdenovo v. 2.5 [62] discovery pipeline. Briefly, this pipeline performs structural and sequence similarity searches to find putative TEs, then performs sequence clustering and functional domain annotation to discard false positives and build a final non-redundant catalog. The final step involves taking into account the domain annotation to classify the TEs by type. The annotation of the elements in the genome was done by running REPET TEannot pipeline on the assembly using the non-redundant catalog, filtering all those elements classified as potential host genes and potential chimeric elements. Short-sequence repeats (SSRs) were annotated in the genome by running RepeatMasker [63]. The resulting GFF3 files belonging to each pipeline were merged to build the final repetitive element catalog, and statistics were obtained with TEannot. The same process was performed for *P. infestans* T30-4 for comparison purposes.

### Gene-dense and gene-sparse genomic architecture analysis

The regions with annotated TEs and SSRs were subtracted from the GFF3 files containing the gene annotation for each assembly using BEDTools v. 2.25.0 [53]. This in order to obtain a file that only included TEs and another file containing only genes that were not part of TEs. The distances among TEs and genes, named flanking intergenic regions (FIRs), were calculated from the resulting GFF3 files using a custom script. We then performed a genomic context analysis using the methodology of Raffaele et al. [8] as implemented by Rojas-Estevez et al. [31]. This analysis aimed to establish a quantitative criterion, the length cutoff (L), to define gene-dense (GDR) and gene-sparse (GSR) regions in both novel assemblies. We first identified single-copy core ortholog clusters using the results obtained in the orthology analysis above. Second, we evaluated different values of L between 100 bp and 20 kbp, and then we computed the percentage of core ortholog genes located in putative GDRs and GSRs relative to the total genes. Third, we calculated the segregation rate defined as the difference between the percentage of all genes in putative GDRs and GSRs, respectively, that are core orthologs. Finally, to select the cutoff L that best classifies the data, we chose the one that maximizes the segregation rate provided that the number of core ortholog genes classified as either gene-dense or in the boundaries of gene-dense regions (in-between) reached saturation (Supplementary Fig. 3).

### Statistics

All statistical analysis and plots were generated using the stats, base, and ggpubr [64] packages of R Statistical Computing Language v. 3.5.1 [65] unless otherwise specified.

## Supplementary information

**Additional file 1**

**Additional file 2**

**Additional file 3**

**Additional file 4**

**Additional file 5**

**Additional file 6**

**Additional file 7**

**Additional file 8**

**Additional file 9**

**Additional file 10**

**Additional file 11**

**Additional file 12**

**Additional file 13**

**Additional file 14**

## Data Availability

The complete datasets including raw sequencing data, the final assemblies, and the annotation features for the genomes generated in this study have been deposited in the BioProject database of the National Center of Biotechnology Information (NCBI) under the accessions PRJNA608953 for *P. betacei* P8084 and PRJNA517953 for *P. infestans* RC1-10. All other datasets generated for this study are included in the article and the supplementary material. Further inquiries can be directed to the corresponding authors.
