# Supplementary figures and images for: A whole genome duplication drives the genome evolution of Phytophthora betacei, a closely related species to Phytophthora infestans
Source: BMC Genomics. 2021 Nov 5;22:795. doi: 10.1186/s12864-021-08079-y (PMC8571832; doi:10.1186/s12864-021-08079-y)

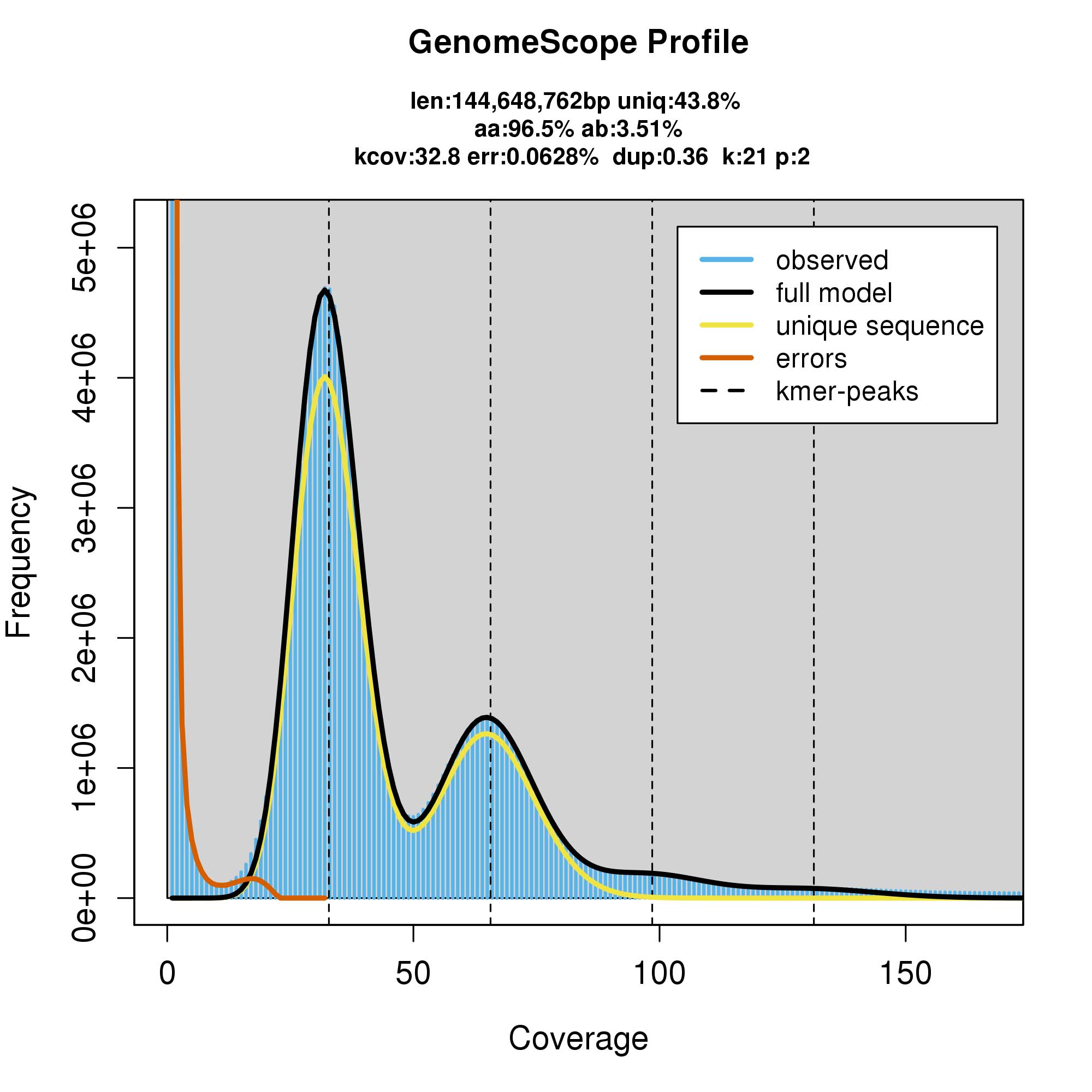

Supplement: Supplementary file 1 — Additional file 1 [file 12864_2021_8079_MOESM1_ESM.jpeg]
